# Supplementary figures and images for: Robustification of RosettaAntibody and Rosetta SnugDock
Source: PLoS One. 2021 Mar 25;16(3):e0234282. doi: 10.1371/journal.pone.0234282 (PMC7993800; doi:10.1371/journal.pone.0234282)

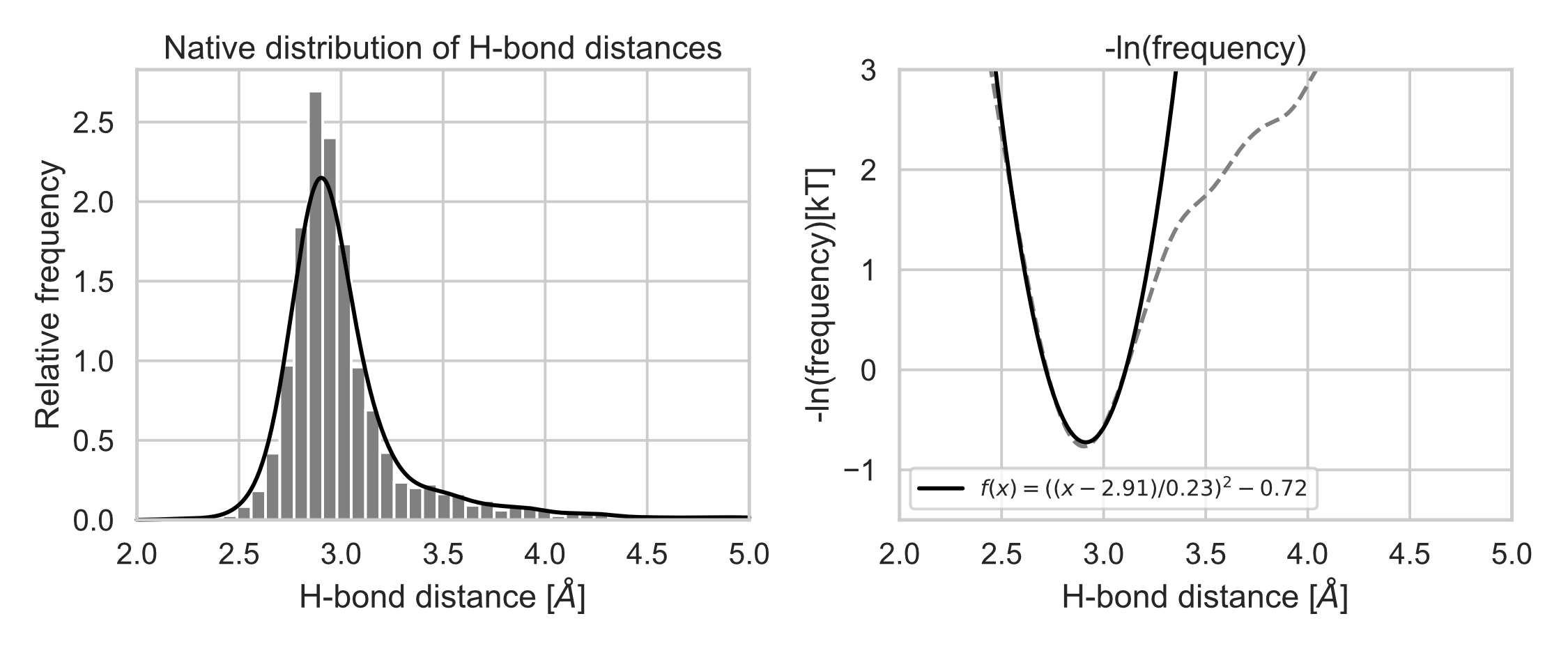

Supplement: S1 Fig — Left: The histogram depicts the observed distances between the oxygen and nitrogen atoms of light chain residue Q38 and heavy chain residue Q39. The distribution was fit by kernel density estimate using Gaussian kernels. Right: The negative logarithm of the probability is proportional to the energy. A harmonic function was fit in the range of 2.5 Å to 3.1 Å. (TIFF) [file pone.0234282.s001.tiff]

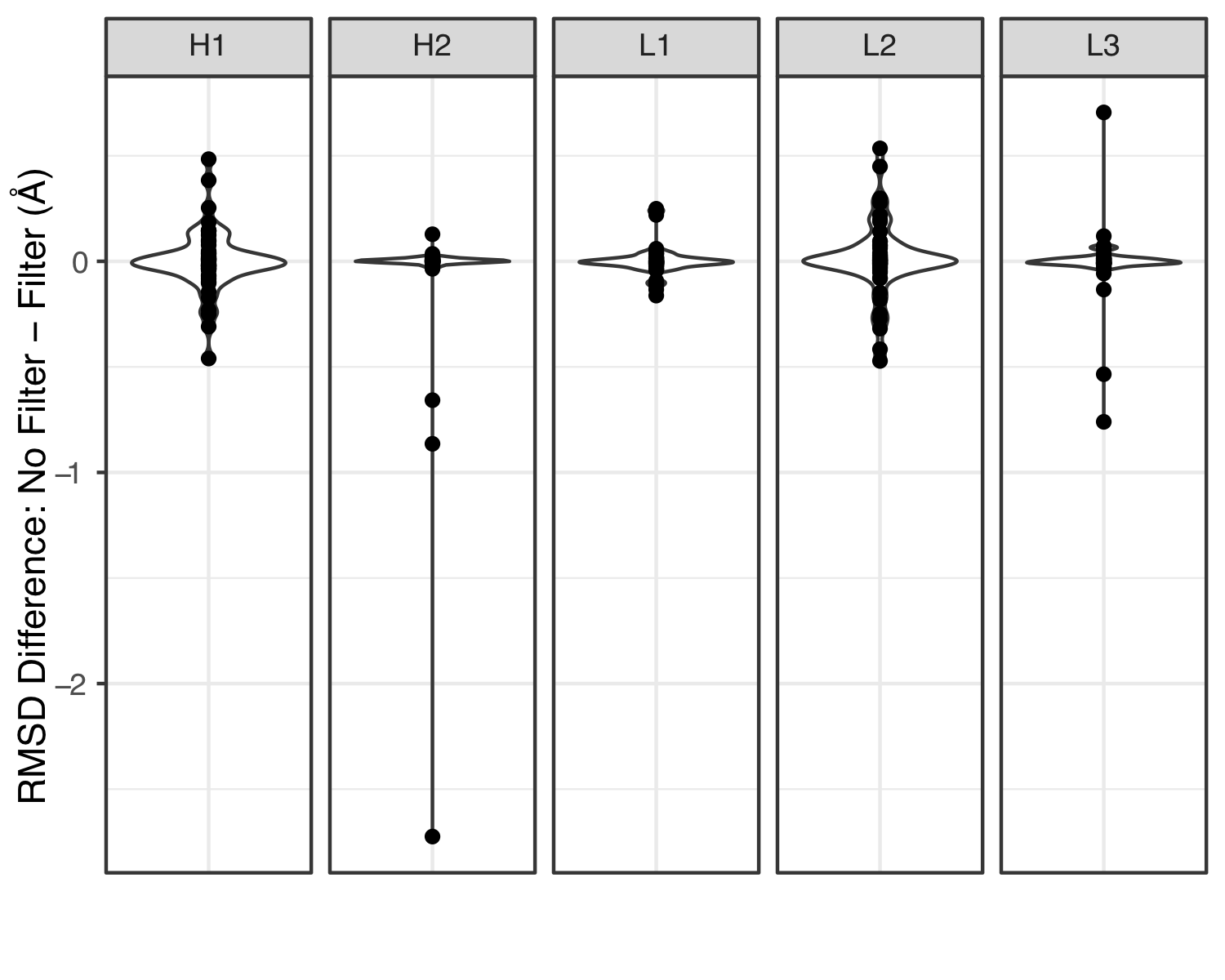

Supplement: S2 Fig — Comparison of the non-H3 CDR loop RMSDs before and after the application of a proline filter. The filter prevents the use of a template when there is a mismatched proline residue with the query. The differences show that most loops are unaffected. In one case for the CDR H2 loop, the loop is model is worse following the application of the filer (moving 2 Å further from the native). This is exclusively due to the presence of an glycine at the start of the target loop (PDB ID: 3LMJ). In the initial model (PDB ID 6EIK, no proline filter), the template also has a glycine, correctly modeling the initial loop structure, whereas the proline-filter-selected template (PDB ID 5LSP) lacks this initial glycine and cannot accurately model the loop start resulting in a cascading worsening of the loop model. All other loops show minor variations within 1 Å. (TIFF) [file pone.0234282.s002.tiff]

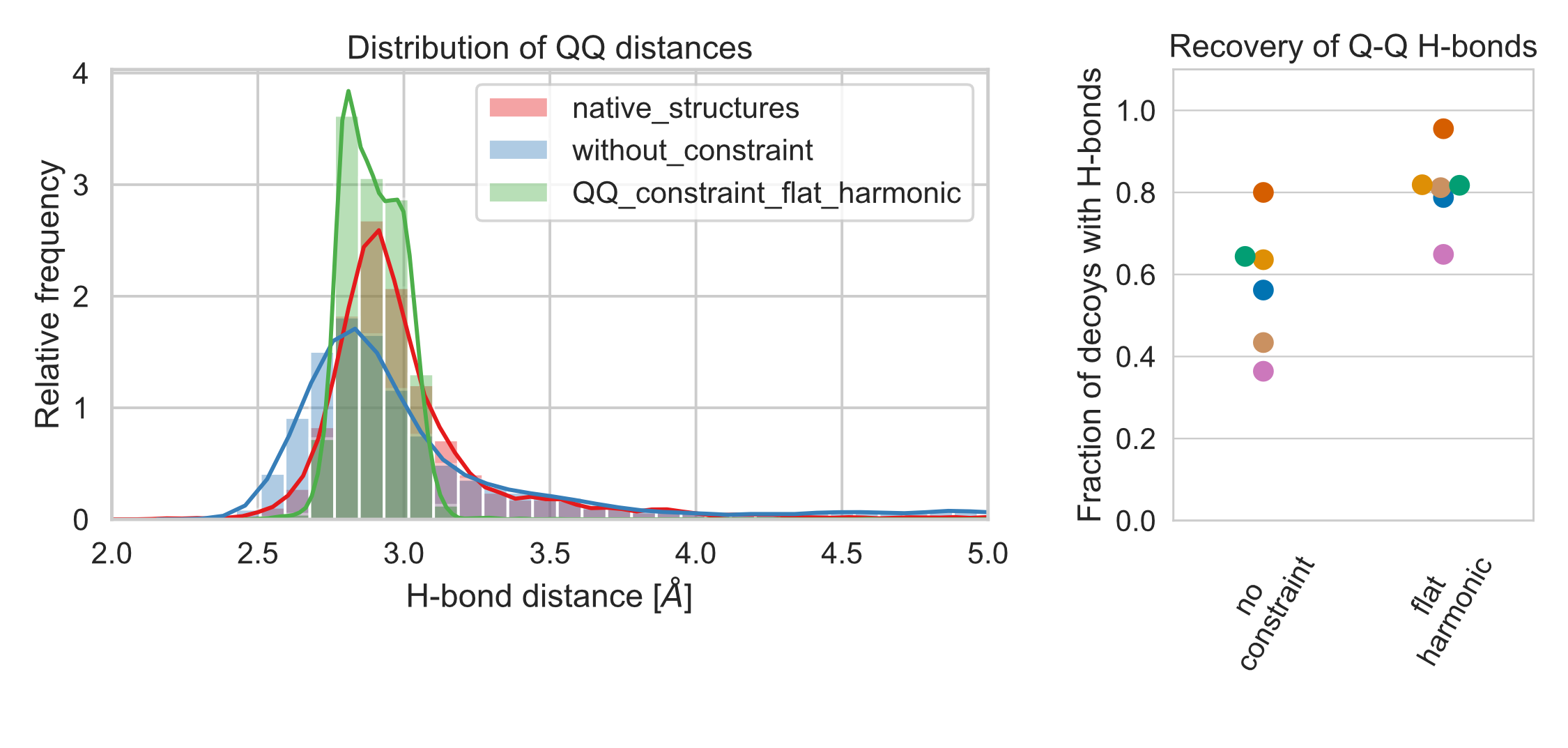

Supplement: S3 Fig — We generated 500 decoys of 6 antibodies with solved structures (S1 Table) either without or with a flat harmonic constraint between the relevant Gln residues. Left: The distances between the nitrogen and oxygen atoms of residues Q38 of the light chain and Q39 of the heavy chain were measured and compared to the native distributions in our antibody database. Right: Each decoy was analyzed for presence of the two possible hydrogen bonds using PyRosetta’s get_hbonds() function. The fraction of decoys forming both hydrogen bonds is shown for each antibody (color-coded). (TIFF) [file pone.0234282.s003.tiff]

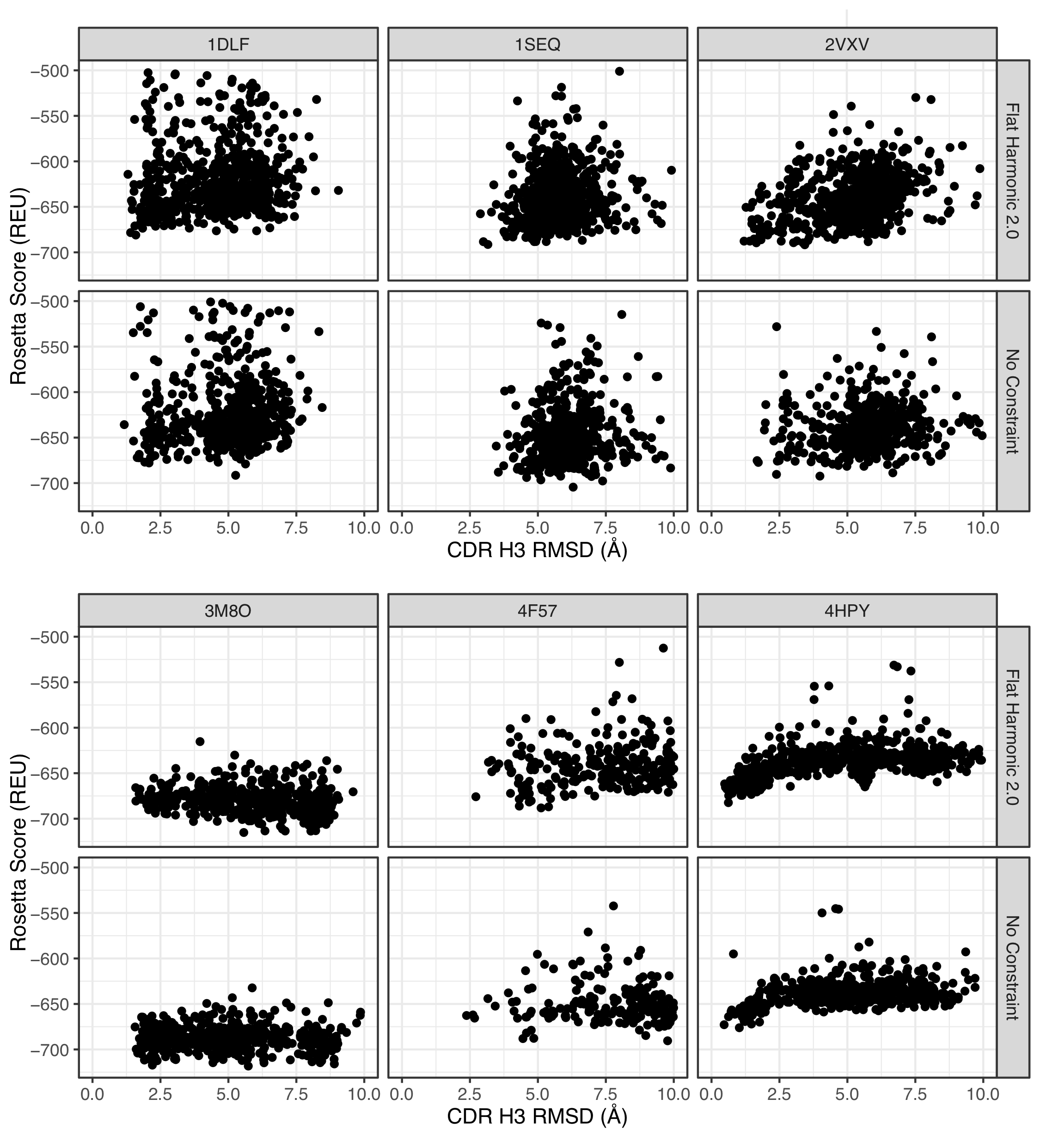

Supplement: S4 Fig — A funnel plot (total score versus CDR-H3 loop RMSD) comparison of RosettaAntibody on six benchmark antibodies does not show a significant difference after the incorporation of the Q–Q constraint. The constraint seemingly improves performance on targets 2VXV and 4F57, but worsens it on 3M8O. (TIFF) [file pone.0234282.s004.tiff]
