# Supplementary material for: Robustification of RosettaAntibody and Rosetta SnugDock
Source: PLoS One. 2021 Mar 25;16(3):e0234282. doi: 10.1371/journal.pone.0234282 (PMC7993800; doi:10.1371/journal.pone.0234282)
Supplement: S1 Table — (PDF) [file pone.0234282.s005.pdf]

S1 Table. Target antibody CDR-H3 loops for the antibody modeling scientific benchmark.

PDB ID	CDR-H3 Length	Difficulty
1DLF	12	Easy
4HPY	13	Easy
2VXV	14	Medium
3M8O	10	Medium
1SEQ	16	Hard
4HPY	18	Hard
