# Supplementary material for: Robustification of RosettaAntibody and Rosetta SnugDock
Source: PLoS One. 2021 Mar 25;16(3):e0234282. doi: 10.1371/journal.pone.0234282 (PMC7993800; doi:10.1371/journal.pone.0234282)
Supplement: S2 Table — (PDF) [file pone.0234282.s006.pdf]

S2 Table. Target antibody–antigen complexes for the docking scientific benchmark.

PDB ID	CDR-H3 Length	Difficulty
1JPS	8	Easy
1MLC	7	Easy
1AHW	8	Medium
1ZTX	10	Medium
2AEP	9	Hard
2JEL	9	Hard
