# Supplementary material for: Robustification of RosettaAntibody and Rosetta SnugDock
Source: PLoS One. 2021 Mar 25;16(3):e0234282. doi: 10.1371/journal.pone.0234282 (PMC7993800; doi:10.1371/journal.pone.0234282)
Supplement: S1 Appendix — (PDF) [file pone.0234282.s007.pdf]

S1 Appendix. List of antibodies used in the grafting benchmark.

```
1dlf,1fns,1gig,1jfq,1jpt,1mfa,1mlb,1mqk,1nlb,1oaq,1seq,2adf,2d7t,
2e27,2fb4,2fbj,2r8s,2v17,2vxv,2w60,2xwt,2ypv,3e8u,3eo9,3g5y,3giz,
3gnm,3go1,3hc4,3hnt,3i9g,3ifl,3liz,3lmj,3m8o,3mxw,3nps,3oz9,3p0y,
3t65,3umt,3v0w,4f57,4h0h,4h20,4hpy,4nzu
```
