# Supplementary material for: Robustification of RosettaAntibody and Rosetta SnugDock
Source: PLoS One. 2021 Mar 25;16(3):e0234282. doi: 10.1371/journal.pone.0234282 (PMC7993800; doi:10.1371/journal.pone.0234282)
Supplement: S2 Appendix — The command line below is used only to compare the grafting assembly stage of RosettaAntibody. In a genuine run, the -no_relax would not be used and -antibody:n_multi_templates 10 would be used instead. (PDF) [file pone.0234282.s008.pdf]

S2 Appendix. RosettaAntibody grafting command line for benchmarking. The command line below is used only to compare the grafting assembly stage of RosettaAntibody. In a genuine run, the `-no_relax` would not be used and `-antibody:n_multi_templates 10` would be used instead.

```
antibody.linuxgccrelease
  -antibody:blastp /path/to/blastp
  -fasta /path/to/input.fasta
  -in:file:native /path/to/xtal.pdb
  -antibody:exclude_pdbs 1ABC
  -antibody:n_multi_templates 1
  -no_relax
```
