# Supplementary material for: Robustification of RosettaAntibody and Rosetta SnugDock
Source: PLoS One. 2021 Mar 25;16(3):e0234282. doi: 10.1371/journal.pone.0234282 (PMC7993800; doi:10.1371/journal.pone.0234282)
Supplement: S5 Appendix — Note constraints are now automatically enabled. To disable constraints, use -antibody:constrain_vlvh_qq false, -antibody:h3_loop_csts_lr false and -antibody:h3_loop_csts_hr false. (PDF) [file pone.0234282.s011.pdf]

S5 Appendix. SnugDock command line. Note constraints are now automatically enabled. To disable constraints, use `-antibody:constrain_vlvh_qq false`, `-antibody:h3_loop_csts_lr false` and `-antibody:h3_loop_csts_hr false`.

```
snugdock.linuxgccrelease
  -s input.pdb
  -partners A_HL
  -nstruct 1000
  -spin
  -dock_pert 3 8
  -detect_disulf false
  -ex1
  -ex2aro
```
