# Supplementary material for: Robustification of RosettaAntibody and Rosetta SnugDock
Source: PLoS One. 2021 Mar 25;16(3):e0234282. doi: 10.1371/journal.pone.0234282 (PMC7993800; doi:10.1371/journal.pone.0234282)
Supplement: S7 Appendix — This will alter the antigen.list and antibody.list files in place. Please note that the chain order in the -partners flag must match the order of chains in the PDB passed by the -s flag and -ensemble1 and -ensemble2. That is to say in the example below the initial_conformation.pdb file has the “A” chain first followed by “H” and “L” while the first ensemble is a list of antigen only structures and the second ensemble is a list of antibody only structures. All structures must have matching numbers of residues. (PDF) [file pone.0234282.s013.pdf]

S7 Appendix. Prepack protocol command line. This will alter the `antigen.list` and `antibody.list` files in place. Please note that the chain order in the `-partners` flag must match the order of chains in the PDB passed by the `-s` flag and `-ensemble1` and `-ensemble2`. That is to say in the example below the `initial_conformation.pdb` file has the "A" chain first followed by "H" and "L" while the first ensemble is a list of antigen only structures and the second ensemble is a list of antibody only structures. All structures must have matching numbers of residues.

```
docking_prepack_protocol.linuxgccrelease
    -s initial_conformation.pdb
    -partners A_HL
    -ensemble1 antigen.list
    -ensemble2 antibody.list
    -ex1
    -ex2aro
    -nstruct 1
```
