# Supplementary material for: Robustification of RosettaAntibody and Rosetta SnugDock
Source: PLoS One. 2021 Mar 25;16(3):e0234282. doi: 10.1371/journal.pone.0234282 (PMC7993800; doi:10.1371/journal.pone.0234282)
Supplement: S8 Appendix — The ensemble of antibody sturctures in this case comes from differ H3 models, but ensembles can also be generated by FastRelax, for example. (PDF) [file pone.0234282.s014.pdf]

S8 Appendix. Sample list file. The ensemble of antibody sturctures in this case comes from differ H3 models, but ensembles can also be generated by FastRelax, for example.

```
antibody_h3_model_1.pdb
antibody_h3_model_2.pdb
antibody_h3_model_3.pdb
antibody_h3_model_4.pdb
antibody_h3_model_5.pdb
```
