# Supplementary material for: Robustification of RosettaAntibody and Rosetta SnugDock
Source: PLoS One. 2021 Mar 25;16(3):e0234282. doi: 10.1371/journal.pone.0234282 (PMC7993800; doi:10.1371/journal.pone.0234282)
Supplement: S9 Appendix — This file only contains two constraints as an example. A complete file would contain one KofNConstraint for each antigen residue with HX-MS data. Each KofNConstraint would contain one flat harmonic constraint for each CDR residue. (PDF) [file pone.0234282.s015.pdf]

S9 Appendix. Sample KofNConstraint file. This file only contains two constraints as an example. A complete file would contain one `KofNConstraint` for each antigen residue with HX-MS data. Each `KofNConstraint` would contain one flat harmonic constraint for each CDR residue.

```
KofNConstraint 1
AtomPair CA 64A CA 24H FLAT_HARMONIC 9 2 1
AtomPair CA 64A CA 25H FLAT_HARMONIC 9 2 1
AtomPair CA 64A CA 26H FLAT_HARMONIC 9 2 1
AtomPair CA 64A CA 27H FLAT_HARMONIC 9 2 1
AtomPair CA 64A CA 28H FLAT_HARMONIC 9 2 1
AtomPair CA 64A CA 29H FLAT_HARMONIC 9 2 1
AtomPair CA 64A CA 30H FLAT_HARMONIC 9 2 1
AtomPair CA 64A CA 31H FLAT_HARMONIC 9 2 1
AtomPair CA 64A CA 32H FLAT_HARMONIC 9 2 1
AtomPair CA 64A CA 39H FLAT_HARMONIC 9 2 1
AtomPair CA 64A CA 40H FLAT_HARMONIC 9 2 1
AtomPair CA 64A CA 41H FLAT_HARMONIC 9 2 1
AtomPair CA 64A CA 42H FLAT_HARMONIC 9 2 1
AtomPair CA 64A CA 57H FLAT_HARMONIC 9 2 1
AtomPair CA 64A CA 58H FLAT_HARMONIC 9 2 1
AtomPair CA 64A CA 59H FLAT_HARMONIC 9 2 1
AtomPair CA 64A CA 60H FLAT_HARMONIC 9 2 1
AtomPair CA 64A CA 64H FLAT_HARMONIC 9 2 1
AtomPair CA 64A CA 65H FLAT_HARMONIC 9 2 1
AtomPair CA 64A CA 66H FLAT_HARMONIC 9 2 1
AtomPair CA 64A CA 67H FLAT_HARMONIC 9 2 1
AtomPair CA 64A CA 68H FLAT_HARMONIC 9 2 1
AtomPair CA 64A CA 69H FLAT_HARMONIC 9 2 1
AtomPair CA 64A CA 107H FLAT_HARMONIC 9 2 1
AtomPair CA 64A CA 108H FLAT_HARMONIC 9 2 1
AtomPair CA 64A CA 109H FLAT_HARMONIC 9 2 1
AtomPair CA 64A CA 110H FLAT_HARMONIC 9 2 1
AtomPair CA 64A CA 111H FLAT_HARMONIC 9 2 1
AtomPair CA 64A CA 112H FLAT_HARMONIC 9 2 1
AtomPair CA 64A CA 113H FLAT_HARMONIC 9 2 1
AtomPair CA 64A CA 114H FLAT_HARMONIC 9 2 1
AtomPair CA 64A CA 115H FLAT_HARMONIC 9 2 1
AtomPair CA 64A CA 116H FLAT_HARMONIC 9 2 1
AtomPair CA 64A CA 117H FLAT_HARMONIC 9 2 1
AtomPair CA 64A CA 118H FLAT_HARMONIC 9 2 1
AtomPair CA 64A CA 119H FLAT_HARMONIC 9 2 1
AtomPair CA 64A CA 120H FLAT_HARMONIC 9 2 1
AtomPair CA 64A CA 121H FLAT_HARMONIC 9 2 1
AtomPair CA 64A CA 122H FLAT_HARMONIC 9 2 1
AtomPair CA 64A CA 123H FLAT_HARMONIC 9 2 1
AtomPair CA 64A CA 137H FLAT_HARMONIC 9 2 1
AtomPair CA 64A CA 138H FLAT_HARMONIC 9 2 1
END
KofNConstraint 1
```

```
AtomPair CA 65A CA 24H FLAT_HARMONIC 9 2 1
AtomPair CA 65A CA 25H FLAT_HARMONIC 9 2 1
AtomPair CA 65A CA 26H FLAT_HARMONIC 9 2 1
AtomPair CA 65A CA 27H FLAT_HARMONIC 9 2 1
AtomPair CA 65A CA 28H FLAT_HARMONIC 9 2 1
AtomPair CA 65A CA 29H FLAT_HARMONIC 9 2 1
AtomPair CA 65A CA 30H FLAT_HARMONIC 9 2 1
AtomPair CA 65A CA 31H FLAT_HARMONIC 9 2 1
AtomPair CA 65A CA 32H FLAT_HARMONIC 9 2 1
AtomPair CA 65A CA 39H FLAT_HARMONIC 9 2 1
AtomPair CA 65A CA 40H FLAT_HARMONIC 9 2 1
AtomPair CA 65A CA 41H FLAT_HARMONIC 9 2 1
AtomPair CA 65A CA 42H FLAT_HARMONIC 9 2 1
AtomPair CA 65A CA 57H FLAT_HARMONIC 9 2 1
AtomPair CA 65A CA 58H FLAT_HARMONIC 9 2 1
AtomPair CA 65A CA 59H FLAT_HARMONIC 9 2 1
AtomPair CA 65A CA 60H FLAT_HARMONIC 9 2 1
AtomPair CA 65A CA 64H FLAT_HARMONIC 9 2 1
AtomPair CA 65A CA 65H FLAT_HARMONIC 9 2 1
AtomPair CA 65A CA 66H FLAT_HARMONIC 9 2 1
AtomPair CA 65A CA 67H FLAT_HARMONIC 9 2 1
AtomPair CA 65A CA 68H FLAT_HARMONIC 9 2 1
AtomPair CA 65A CA 69H FLAT_HARMONIC 9 2 1
AtomPair CA 65A CA 107H FLAT_HARMONIC 9 2 1
AtomPair CA 65A CA 108H FLAT_HARMONIC 9 2 1
AtomPair CA 65A CA 109H FLAT_HARMONIC 9 2 1
AtomPair CA 65A CA 110H FLAT_HARMONIC 9 2 1
AtomPair CA 65A CA 111H FLAT_HARMONIC 9 2 1
AtomPair CA 65A CA 112H FLAT_HARMONIC 9 2 1
AtomPair CA 65A CA 113H FLAT_HARMONIC 9 2 1
AtomPair CA 65A CA 114H FLAT_HARMONIC 9 2 1
AtomPair CA 65A CA 115H FLAT_HARMONIC 9 2 1
AtomPair CA 65A CA 116H FLAT_HARMONIC 9 2 1
AtomPair CA 65A CA 117H FLAT_HARMONIC 9 2 1
AtomPair CA 65A CA 118H FLAT_HARMONIC 9 2 1
AtomPair CA 65A CA 119H FLAT_HARMONIC 9 2 1
AtomPair CA 65A CA 120H FLAT_HARMONIC 9 2 1
AtomPair CA 65A CA 121H FLAT_HARMONIC 9 2 1
AtomPair CA 65A CA 122H FLAT_HARMONIC 9 2 1
AtomPair CA 65A CA 123H FLAT_HARMONIC 9 2 1
AtomPair CA 65A CA 137H FLAT_HARMONIC 9 2 1
AtomPair CA 65A CA 138H FLAT_HARMONIC 9 2 1
END
```
