# Supplementary material for: Robustification of RosettaAntibody and Rosetta SnugDock
Source: PLoS One. 2021 Mar 25;16(3):e0234282. doi: 10.1371/journal.pone.0234282 (PMC7993800; doi:10.1371/journal.pone.0234282)
Supplement: S10 Appendix — Additional constraints can be added to both the low- and high-resolution stages of SnugDock. MDS is a special score function for the low-resolution stage of docking. It has been found to improve performance in protein–protein complex docking. It can be used in SnugDock as well. (PDF) [file pone.0234282.s016.pdf]

S10 Appendix. SnugDock command line with constraints and motif dock score (MDS). Additional constraints can be added to both the low- and high-resolution stages of SnugDock. MDS is a special score function for the low-resolution stage of docking. It has been found to improve performance in protein–protein complex docking. It can be used in SnugDock as well.

```
snugdock.linuxgccrelease
  -s initial_conformation.pdb
  -partners A_HL
  -ensemble1 antigen.list
  -ensemble2 antibody.list
  -nstruct 1000
  -spin
  -dock_pert 3 8
  -detect_disulf false
  -ex1
  -ex2aro
  -constraints:cst_fa_file high-res.cst
  -constraints:cst_file low-res.cst
  -docking_low_res_score motif_dock_score
  -mh:path:scores_BB_BB /path/to/motif_dock/score_data_
  -mh:score:use_ss1 false
  -mh:score:use_ss2 false
  -mh:score:use_aa1 true
  -mh:score:use_aa2 true
```
