# Supplementary material for: Robustification of RosettaAntibody and Rosetta SnugDock
Source: PLoS One. 2021 Mar 25;16(3):e0234282. doi: 10.1371/journal.pone.0234282 (PMC7993800; doi:10.1371/journal.pone.0234282)
Supplement: S11 Appendix — Exemplary flags for global docking with constraints. (PDF) [file pone.0234282.s017.pdf]

S11 Appendix. Global docking command line. Exemplary flags for global docking with constraints.

```
docking_protocol.linuxgccrelease
  -s initial_complex.pdb
  -nstruct 1000
  -spin
  -partners H_A
  -randomize1
  -randomize2
  -use_ellipsoidal_randomization true
  -detect_disulf true
  -rebuild_disulf true
  -ex1
  -ex2aro
  -constraints:cst_file low-res.cst
  -constraints:cst_fa_file high-res.cst
```
